# Reaching consensus amongst international experts on the use of high importance-rated antimicrobials in animals – a Delphi study

**DOI:** 10.1016/j.onehlt.2024.100883

**Published:** 2024-08-26

**Authors:** Anna Sri, Kirsten E. Bailey, Ri Scarborough, James R. Gilkerson, Karin Thursky, Glenn F. Browning, Laura Y. Hardefeldt

**Affiliations:** a Asia-Pacific Centre for Animal Health, Melbourne Veterinary School, Faculty of Science, University of Melbourne, Parkville, VIC 3010, Australia; b National Centre for Antimicrobial Stewardship, Department of Infectious Diseases Melbourne Medical School and Melbourne Veterinary School, University of Melbourne, VIC 3010, Australia

**Keywords:** Antimicrobial resistance, High-priority critically important, Antimicrobial stewardship, Rating systems, One health

## Abstract

In Australia, antimicrobials are given an importance rating by the Australian Strategic and Technical Advisory Group on antimicrobial resistance. High importance antimicrobials are those essential for the treatment or prevention of infections in humans, where there are few or no treatment alternatives. In this study we consulted with experts from across human and animal health using the Delphi consensus-building process to establish the circumstances under which antimicrobials with high importance to human health could be used in animals in Australia. We used three rounds of online surveys. Group responses were provided to participants in each subsequent round to facilitate convergence of opinion. Consensus was defined as 80 % or more of respondents selecting the same option for a question. By the end of the third round, consensus was achieved on eight items. This included the use of high importance antimicrobials being appropriate if culture and sensitivity testing indicated the organism was resistant to low- and medium-rated antimicrobials that could be used to treat the case. If any high-importance antimicrobials are prescribed for animals there was also agreement that a clear indication for this use and justification for antimicrobial choice must be recorded in the medical history, along with the dose rate, route of administration, the duration and the time point for review of the condition and associated antimicrobial therapy.

Appropriateness of use of high importance antimicrobials in critically ill animals where culture and sensitivity results are not available is still undefined. Further work is also required to establish which particular organisation should be notified of the use of high importance antimicrobials not registered for use in animals. The Delphi process was valuable in facilitating consensus amongst international experts from a broad range of health backgrounds and experience.

## 1 Introduction

The Delphi consensus method is a group facilitation process used to provide expert guidance about the best course of action to take when faced with a problem or issue for which there is no clear solution [1]. The Delphi process involves administration of rounds of surveys and provision of anonymous feedback to participants with the aim of identifying areas where consensus can be reached. It is useful when participants are geographically dispersed and anonymity is desired to control for individuals whose opinions could dominate or disproportionately influence others [2]. The process has been used in numerous contexts to broaden knowledge and advance practice, particularly in relation to guideline development and establishing relevant metrics, for example, for measuring appropriateness of prescribing [[3], [4], [5]]. It is particularly useful where higher quality evidence may not be available [6] or when current evidence may be contradictory but some agreement on the way forward would be beneficial [2].

Currently, such a situation exists in the field of antimicrobial stewardship, particularly about the future consequences of current actions. There is widespread acknowledgement that antimicrobial use in animals needs to be recognised as a contributor towards antimicrobial resistance (AMR) [[7], [8], [9]], but also that veterinarians' access to antimicrobials is essential to treat infectious disease and maintain good animal welfare [10,11]. In Australia, antimicrobials are assigned an importance rating by the Australian Strategic and Technical Advisory Group on antimicrobial resistance (ASTAG) [12]. The ASTAG rating system is based upon, and considers, the World Health Organisation's Critically Important Antimicrobials list. However, ASTAG has rated some antibiotics differently, depending on the local situation in Australia, where the prevalence of resistance and disease in humans and animals differs from other parts of the world [13,14]. High importance antibacterials are those “essential for the treatment or prevention of infections in humans where there are few or no treatment alternatives for infections”. Use of these drugs in animals is contentious as it could contribute towards the development of antimicrobial resistance that may impact the effective treatment of human infections.

The aim of this study was to define the circumstances under which antimicrobials of high importance in human health could be used in animals. Achieving consensus amongst medical and veterinary experts on key elements of antimicrobial use and stewardship will facilitate progress by enabling a unified approach to the use and management of this shared resource. It will also demonstrate that all sectors can work together to promote antimicrobial stewardship and slow the progression of antimicrobial resistance. For the purposes of this study, antimicrobial stewardship was defined both specifically, as activities that reduce the development of AMR in a clinical setting [15], and also more broadly, as a concept or multifaceted approach that has the ultimate goal of reducing pressure on the development of AMR [16,17].

## 2 Materials and methods

Potential participants were identified through the researchers' professional networks and by searching for published authors in the field of AMS. Our research group includes veterinarians and physicians with expertise in infectious disease, microbiology, antimicrobial resistance, antimicrobial stewardship, and public health.

Questions were formulated based on identified gaps in previous research [9,18], with the aim of establishing the circumstances under which antimicrobials of high importance in human health could be used in animals (or if such circumstances ever exist). Issues such as insufficient detail in medical records to assess appropriateness of use were also explored. Reference to previous research also identified areas where there was already significant agreement, such as about the use of culture and sensitivity testing [19]. Questions covered a variety of topics, including establishing the area and years of expertise of the participants, importance rating systems, as well as questions about different situations in which use should be restricted – areas currently lacking sufficient Australian-specific research. Ample opportunity was provided for free-text comments and explanation of the participant's reasoning, and these informed the wording of questions in subsequent rounds and the provision of additional information. Questions were presented back to the group in later rounds after adjustments to the focus or wording based on feedback from earlier rounds. The full text of each survey can be found in the supplementary materials.

English-speaking experts in the field of AMR or AMS were invited to participate by email. Expertise was defined as: having published in the field of AMR or AMS; membership of a working group, committee or other organisation addressing AMR, AMS or a related discipline; or working in a relevant field (e.g. infectious disease, microbiology, AMS, AMR, IPC, medicine, surgery, or public health). Many of the participants had published work that members of the research group were aware of and were thus acknowledged as ‘experts’ in their field. The participants' level of expertise and involvement in AMS was also established in the first survey round. Three survey rounds were undertaken online between November 2021 and May 2022.

Consensus was pre-defined as 80 % or more of respondents selecting either the same option or a similar option. This level of consensus was chosen as it represented a considerable majority of participants, ensuring acceptance of these results amongst human and animal health experts, particularly when advocating for changes in policy or usual practice. Of note is that there was a trend towards consensus and stability of results throughout the rounds, which has been reported as being important in addition to absolute percentage of consensus [20]. Research on defining appropriate levels of consensus for Delphi studies is limited [4,20]. The Delphi process involved reporting results from each survey round in the subsequent round and asking experts to evaluate their responses based on the provision of additional clarification or information and on the responses of the group as a whole. In subsequent rounds, options such as ‘agree’ and ‘strongly agree’ or ‘disagree’ and ‘strongly disagree’ were consolidated to simplify choices, encouraging convergence of opinion. Questions and topics were excluded if no option was within 15–20 % of consensus or the comments suggested a need for extensive discussion or engagement to achieve agreement, beyond what could be achieved solely through additional survey rounds.

## 3 Results and discussion

Surveys were distributed to 119 experts across human and veterinary medicine. The response rate to the surveys was 38 % for Round 1, with 45 experts responding (although one response was only partially completed). Forty-eight percent of respondents (*n* = 21) prescribed antibiotics in their current role (Table 1), 86 % of whom were veterinarians (*n* = 18). Eighteen (41 %) of the experts who were the lead of an antimicrobial stewardship team, or who had executive oversight over an antimicrobial stewardship team (*n* = 25, 57 %), were also veterinarians. Most participants (*n* = 38, 86 %) had been working in antimicrobial stewardship or antimicrobial resistance for over 5 years, and fourteen respondents (32 %) had over twenty years of experience in these fields. For the second and third rounds, the survey was sent to the Round 1 respondents and response rates were 53 % (24/45) and 56 % (25/45) respectively. Seventy-five percent of respondents that took part in Round 2 also took part in Round 3 (18/24). The Australian and international experts had a diverse range of occupations and areas of expertise (Table 1).

**Table 1:** Participant characteristics.

	Round 1	Round 3
Characteristic	n (%)	n (%)
Total number of participants	44/119	25/45
Prescriber		Not asked
Yes	21 (48)	
No	23 (52)	

Lead or oversight of AMS team		Not asked
Yes	25 (57)	
No	19 (43)	

Occupation		
Academic	1 (2.3)	4 (16)
Epidemiologist	1 (2.3)	1 (4)
Veterinarian and academic	1 (2.3)	6 (24)
Veterinarian and microbiologist	3 (6.8)	–
Veterinarian	23 (52)	8 (32)
Veterinarian and consultant	1 (2.3)	–
Physician/surgeon	2 (4.5)	–
Physician/surgeon and microbiologist	1 (2.3)	1 (4)
Pharmacist	2 (4.5)	1 (4)
Medical or veterinary microbiologist	5 (11)	2 (8)
Government employee	1 (2.3)	1 (4)
Employed in industry/pharmaceutical sector	3 (6.8)	–
Other	–	1 (4)

Species worked with predominantly (for veterinarians only)		Not asked
Dog and cats	8 (18)	
Horses	2 (4.5)	
Dogs, cats, horses	2 (4.5)	
Dogs, cats, horses, exotics	1 (2.3)	
Dairy cattle	4 (9.1)	
Poultry	2 (4.5)	
Pigs	2 (4.5)	
Sheep	1 (2.3)	
Other	1 (2.3)	
All Species	7 (16)	
None	14 (32)	

Area of Expertise*		Not asked
Infectious diseases	27 (61)	
Microbiology	19 (43)	
Antimicrobial stewardship	32 (73)	
Antimicrobial resistance	26 (59)	
Infection prevention and control	19 (43)	
Medicine	13 (30)	
Dermatology	1 (2.3)	
Surgery	3 (6.8)	
General practice	8 (18)	
Epidemiology	2 (4.5)	
Zoonoses, food safety	1 (2.3)	
Regulation of veterinary medicines	2 (4.5)	

Years working in antimicrobial stewardship or resistance		Not asked
< 5	6 (14)	
5–10	16 (36)	
11–19	8 (18)	
>20	14 (32)	

Country		
Australia	27 (61)	14 (58)
Brazil	1 (2.3)	–
Canada	3 (6.8)	1 (4.2)
Denmark	1 (2.3)	1 (4.2)
Netherlands	1 (2.3)	1 (4.2)
New Zealand	1 (2.3)	1 (4.2)
Sweden	1 (2.3)	–
United Kingdom	5 (11)	3 (13)
United States	4 (9.1)	3 (13)
Not answered	–	1 (4.2)

NB: Participant characteristic data was not collected in round 2 to minimise survey length.

**Fig. 1:**
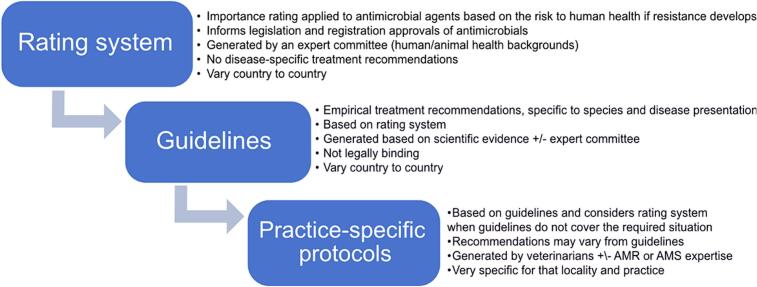
Definitions provided to participants in Delphi survey Round 2.

In Round 1, most participants (*n* = 28, 64 %) indicated they used a country-specific antimicrobial importance rating system in their practice. This correlated with responses to a subsequent question, which asked participants for their view about which rating systems veterinarians *should* use, with 73 % (32/44) of respondents selecting country-specific ratings. However, the reasoning behind these responses suggested there were varying levels of understanding and definitions used for rating systems, guidelines and practice-specific antimicrobial use policies. Therefore, participants in Round 2 were provided with a flow chart (Fig. 1) to more clearly define these terms and how they related to each other. This resulted in consensus in Round 2, where 96 % of respondents (*n* = 23) agreed ‘*The country-specific rating system should take precedence over any other rating system (e.g. WHO rating system) when veterinarians make decisions about antimicrobial prescribing choices’*. There was also consensus (*n* = 20, 83 % agreement) that ‘*Veterinarians should be able to create local practice-specific antimicrobial use protocols, but should not be able to create their own practice-specific antimicrobial importance rating systems’*. This consensus item was important as it removed the capacity for veterinarians to justify inappropriate use based on a less robust importance rating system.

Restricting antimicrobial use can reduce selection for antimicrobial resistance [9]. While education is an essential part of any AMS plan, controlling antimicrobial use in human and animal populations through restrictions or system-level interventions may have more impact than raising public or practitioner awareness [21]. The low levels of resistance to fluoroquinolones in Australia, which is associated with a long history of prohibiting the use of this class of antimicrobials in food-producing animals, seems to support the beneficial effect of restricting antimicrobial use [7].

**Fig. 2:**
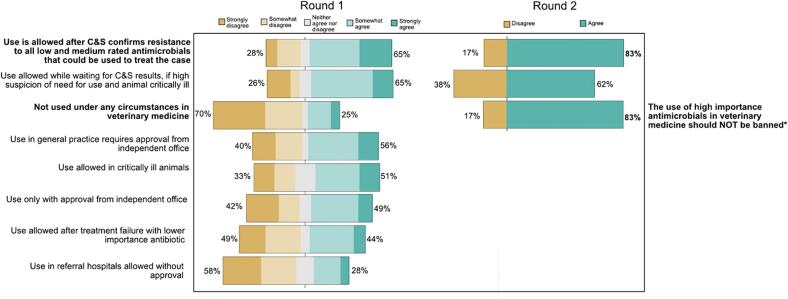
Participants' responses to the question *‘If there were restrictions placed on veterinary prescribing of high-importance antimicrobials, which of the following do you think is appropriate?* Consensus was reached on the items in bold. The item marked with an asterisk was adapted for Round 2.

Participants were asked to identify antimicrobials to which they thought restrictions on use should apply. Seven possible options were provided: all antimicrobials, all antimicrobials with a medium- or high-importance rating, all antimicrobials with a high importance rating, all antimicrobials with a high-importance rating except for third generation cephalosporins and fluoroquinolones, all antimicrobials with a high-importance rating except for fluoroquinolones, all antimicrobials with a high-importance rating except for third generation cephalosporins, or no antimicrobials should be restricted. There were diverse views on this topic. A higher proportion of people selected ‘All antimicrobials with a high-importance rating” in the second round (*n* = 17, 71 %) after viewing the results of Round 1 (*n* = 21, 48 %). Less popular options were also removed for Round 2, but consensus could still not be reached. In Round 1, participants were asked how strongly they agreed or disagreed with a range of possible restrictions. In Round 2 only the most popular restriction conditions were retained, and response options provided were limited to agree or disagree (Fig. 2).

Agreement was reached that, “*Use of high importance antimicrobials is allowed after culture and susceptibility testing confirms that the pathogen is resistant to all low- and medium-rated antimicrobials that could be used to treat the case*” (*n* = 20, 83 %). This option also consistently received high levels of support in a previous survey of Australian veterinarians, but concerns around costs and timeliness of results were raised [19]. A participant in our study also mentioned the “*widespread confusion with regards to how to interpret susceptibility data and what it really means*” and that this could be leading to incorrect prescribing decisions. This suggests further research, as well as educational, policy and financial support, is required to address concerns related to the costs of culture and susceptibility testing, its accessibility and the waiting times for results, particularly for regional and remote areas, and to ensure the correct prescribing actions are taken by veterinarians on receiving culture and susceptibility testing results [19,22,23].

While there was support for the option to allow use of high importance antimicrobials for critically ill patients (Fig. 2), the practicalities of defining the subjective terms ‘critically ill’ and ‘high suspicion of need’, led to deletion of this option from round 3. It is important to note that welfare concerns were raised by a number of participants, particularly for critically ill patients (see comments 2a and 2b in Appendix A, Table 2), consistent with previous research findings [19,21]. The appropriate approach in this scenario will need to be explored further during any implementation of these consensus stewardship measures to ensure there are no unintended consequences [11].

**Table 2:** Importance of reducing high-importance antimicrobial use.

Question	Responses (%)				
	Very Important	Important	Moderately Important	Slightly Important	Not Important
*How important do you think it is to reduce high-importance antimicrobial use in companion animals?*	15 (60)	7 (28)	3 (12)	0 (0)	0 (0)
*How important do you think it is to reduce high-importance antimicrobial use in food-producing animals?*	18 (72)	5 (20)	2 (8)	0 (0)	0 (0)

Participants were asked whether antimicrobials with high importance in human medicine that are registered for use in veterinary medicine (such as third generation cephalosporins and fluoroquinolones) should be treated differently to those only registered for use in humans. The majority of participants answered ‘yes’ (*n* = 26, 59 %) with 14 % (*n* = 6) ‘unsure’ and 27 % (*n* = 12) answering ‘no’. The results and free text comments led to a refined statement, which achieved consensus (*n* = 20, 83 %), *“Any use of high importance antimicrobials that are not registered for use in animals, e.g. vancomycin, amikacin or imipenem, must be reported to a central authority.”* Surveillance of and understanding about the use of high importance antimicrobials not registered for use in animals is lacking. This makes assessing the quality of this prescribing difficult and also leads to gaps in our knowledge. In their reasoning for supporting this restriction condition, a number of participants highlighted the value of reporting use, including for educational purposes, identifying gaps for future research, and making prescribers more accountable (see example quotes 4a, 4b, 4c, 4d and 4e in Appendix A, Table 4).

Consensus was not established about which central authority should receive these reports. Based on the free text responses from the previous round, explanatory text was provided to participants (see Supplementary materials). An option to report to a “*newly created independent federal body to address antimicrobial resistance using a One Health framework (e.g. Centre for Disease Control type of organisation or a One Health central authority)*” was provided in the subsequent survey round and was the most popular option. However, this option was still only selected by 36 % (*n* = 5) of respondents, followed by the State Department of Health (21 %, *n* = 3) and the State Department of Agriculture (21 %, n = 3). There was little support for reporting to the office of the Chief Veterinary Officer (OCVO) (8 %, *n* = 2), the Australian Strategic and Technical Advisory Group (ASTAG) on AMR (0 %, *n* = 0), or the Australian Pesticides and Veterinary Medicines Authority (0 %, n = 0).

There was no consensus about who should provide independent approval to use a restricted antimicrobial, but the greatest support was for a practice stewardship champion (43 %) or a veterinary microbiologist (41 %). Participants stated that whoever provided approval for use would need to be objective and independent, have a broad range of knowledge and skills (including, but not limited to, microbiology, pathophysiology, public health, and AMS), and potentially this approval would be best sought from a team of experts (see quotes 3a, 3b, 3c and 3d in Appendix A, Table 3).

There was consensus that clarified details about reporting and medical records. Previous research has found veterinary medical records are often insufficiently complete to make a judgement about the quality of prescribing and that this is an area that could be improved [24]. For example, medical records frequently lack information about the duration of antimicrobial use, the dose rate prescribed, and the indication for use [[25], [26], [27]].

Reducing the use of high-importance antimicrobials in both companion animals and food-producing animals was important or very important to most participants (88 % and 92 %, respectively; Table 2). The need to reduce antimicrobial use has received less focus in companion animal medicine than in food-producing animal medicine, in both research and regulation, but it is an emerging area of concern. Many people are very close with their companion animals and the risk of transfer of resistant bacteria or antimicrobial resistance genes from animals to humans, or vice versa, has been identified [28,29]. Currently in Australia there are fewer restrictions on the use of high-importance antimicrobials in companion animals, including horses. As a result, use of high-importance antimicrobials in these species is much more common than in food-producing animals [24,27,30,31].

Several participants indicated they would have preferred to answer some questions differently for companion animals and food-producing animals. While there are differences between these differing animal populations in the risks associated with the development of antimicrobial resistance, we decided not to differentiate between them in most questions about use in domestic animals, given the increasing emergence of antimicrobial resistance in companion animals in Australia [32]. Continually focusing only on the role of food-producing animals in disseminating or amplifying AMR may delay and detract from antimicrobial stewardship practices in the companion animal health sector because use in this sector is perceived to be a less important driver of AMR.

### 3.1 Consensus statements

In total, consensus was reached amongst participants on eight items relating to rating systems and the use of high importance antimicrobials.

1. Reducing the use of high-importance antimicrobials in companion animals and food-producing animals is important.
2. The country-specific rating system should take precedence over any other rating system (e.g. WHO-rating system) when veterinarians make decisions about antimicrobial prescribing choices.
3. When using international prescribing guidelines, these should be adapted to account for the country-specific rating system. e.g. International Society for Companion Animal Infectious Diseases (ISCAID), British Equine Veterinary Association (BEVA).
4. Veterinarians should be able to create local practice-specific antimicrobial use protocols but should not be able to create their own practice-specific antimicrobial importance rating systems.
5. Use of high-importance antimicrobials is allowed after culture and susceptibility testing confirms the pathogen is resistant to all low- and medium-rated antimicrobials that could be used to treat the case.
6. The use of high-importance antimicrobials in veterinary medicine should NOT be banned.
7. Any use of high-importance antimicrobials not registered for use in animals must be reported to a central authority. Examples include vancomycin, amikacin or imipenem.
  a. The purpose of the reporting should be for record keeping over time for surveillance of antimicrobial use and for auditing or investigating high or frequent users to assist users in finding ways to reduce unnecessary use (non-punitive).
  b. The species the antimicrobial has been prescribed for should be reported along with any other justification, such as supporting diagnostic tests, the reasons other lower importance antimicrobials could not be used, and whether culture and sensitivity testing has been used previously to support the use of the high-importance antimicrobial.
8. If any high-importance antimicrobials are prescribed for animals, a clear indication for use and justification for antimicrobial choice must be recorded in the medical history, along with the dose rate, route of administration, the duration and the time point for review of the condition and associated antimicrobial therapy.

Participants in this study were provided the opportunity to include free-text comments to explain their reasoning. The option for participants to provide this clarification counteracted some of the concerns that can arise with the Delphi process, such as insufficient opportunity to explore participants' underlying assumptions [33]. In our case, the ability to identify areas of concern facilitated the provision of further information on numerous occasions, ultimately leading to development of consensus.

Some of the free text comments demonstrated a lack of knowledge about how rating systems are determined and developed, even amongst experts. There is a need for more education about the process for generating rating systems to facilitate compliance, as, even amongst experts, levels of understanding differed (see free text comments 1a, 1b, 1c, and 1d in Appendix A, Table 1). Some current divergence within or between professions and sectors may result from varying levels of comprehension, misunderstandings, and different definitions, rather than from completely contrasting viewpoints. On a small number of occasions, participant's responses to the survey question contradicted the reasoning provided in the free text comments. In these cases, the capacity for in-person clarification and discussion would have been beneficial.

This study builds on the results of a previous study of Australian veterinarians [19] that showed veterinarians mostly agreed with restrictions on the use of high importance antimicrobials in animals, particularly on the need for evidence of AMR from culture and sensitivity testing for use of high-importance antimicrobials to be considered reasonable [19]. The situation in Australia is markedly different from countries where antimicrobials are widely available without a prescription [34]. While veterinarians, like medical doctors, are wary of too much involvement, particularly of bureaucrats and non-veterinarians, in their practice and decision-making [35], a balance needs to be found between restricting use for future benefit and enabling the timely and effective treatment of animals when they need it [9].

The rates of participation of human health professionals in this study were lower than that of veterinarians. While it is not possible to determine whether there was less interest in a One Health approach amongst human health experts, participation may have been impacted by the requirement for human health experts at that time to focus on the COVID-19 pandemic response. Other research has shown a decrease in focus on AMS during the COVID-19 pandemic [36].

The high response rate over multiple rounds, as well as the robust reasoning provided by participants in free-text comments, strengthened the validity of the consensus items developed throughout this process. The use of anonymity, which ensured that dominant individual viewpoints did not overshadow group consensus, as well as the engagement of experts with extensive histories working in the field of antimicrobial stewardship supports the validity of our results.

## 4 Conclusions

The use of the Delphi process proved effective in seeking consensus about appropriate restrictions on use of antimicrobials in animal health, despite the additional pressures on participants during the COVID-19 pandemic and was enhanced by the inclusion of experts with a wide range of skills and expertise from geographically diverse areas. Consensus on numerous key indicators suggests paths forward to advance antimicrobial stewardship in animal health in Australia. This study provides veterinarians clear guidance in their daily practice about deciding whether a current clinical situation warrants the use of a high-importance antimicrobial. There was clear agreement about the need to justify, record and report any use of high-importance antimicrobials. Suggestions directly relevant to policy development have been made and will require input from both the public and private sectors. It is hoped these consensus items, identified through a robust process involving a variety of international and national experts will be helpful for practicing veterinarians and policymakers alike in management of antimicrobials as a shared resource.

## Ethics approval

The study was conducted according to the guidelines of the Declaration of Helsinki, and approved by the Human Research Ethics Committee of the University of Melbourne (protocol code:13824, date of approval: 4th August 2020).

## Informed consent statement

Informed consent was obtained from all subjects involved in the study.

## Funding

This study was funded by the Australian Research Council through the Discovery Early Career Research Award program DE200100030 awarded to L.Y.H. A.S. and R.S. were the recipients of Australian Postgraduate Award scholarships. K.B. was supported through NHMRC CRE Application 1079625.

## CRediT authorship contribution statement

**Anna Sri:** Conceptualization, Data curation, Formal analysis, Investigation, Methodology, Visualization, Writing – original draft, Writing – review & editing. **Kirsten E. Bailey:** Conceptualization, Methodology, Supervision, Writing – review & editing. **Ri Scarborough:** Conceptualization. **James R. Gilkerson:** Conceptualization, Supervision, Writing – review & editing. **Karin Thursky:** Conceptualization. **Glenn F. Browning:** Conceptualization, Writing – review & editing. **Laura Y. Hardefeldt:** Conceptualization, Funding acquisition, Methodology, Supervision, Writing – review & editing.

## Declaration of competing interest

The authors declare that they have no known competing financial interests or personal relationships that could have appeared to influence the work reported in this paper.

## Figures and Tables

**Table 1:** Sample of free text responses about rating systems for antimicrobial importance in veterinary medicine.

Reference	Quote
1a	There is a real need for unity in this area - having so many ratings systems all disagreeing with each other confuses prescribers and lowers the impact of such effects. Although I have selected WHO here, in reality I am more pointing towards the need for a single, internationally agreed approach adopted by all countries, whether that would or would not be led by WHO.
1b	Ratings systems should be based on an AM's importance to human medicine and risks of AMR development, not on factors such as cost, convenience, etc.
1c	Rating systems in veterinary medicine should align with actual evidence rather than theoretical hysterical hyperbole. In other words, does the evidence justify the classification. (e.g. 1. evidence of actual veterinary/human AMR issues being proven to be the result of specific drug usage in veterinary practice over time).
1d	More harmony, or at least removing inconsistency, between country rating systems would be helpful. Also more accessible explanation or methodology on how importance ratings are decided.

**Table 2:** Free-text responses about how high-importance antimicrobials that are registered for use in veterinary medicine be treated differently to those that are not registered use in veterinary medicine.

Reference	Quote
2a	Only use when lower importance products are unlikely to be effective or when high risk of poor patient outcome likely if alternative antimicrobial is used. In food producing animals the antimicrobial spectrum and alternatives is sometimes limited relative to the anticipated pathogen. In cases such as a septic neonate waiting for the antimicrobial susceptibility may have dire consequences for the patient.
2b	Use should be on strict guidelines which require substantiation and review by the prescribing veterinarian. For example: Emergency, short-term use of virginiamycin to grazing ruminants should be permitted on animal welfare grounds under specified protocols and/or guidelines for adaptation to grain feeding such as under circumstances of drought or pasture toxicity. However, prescribing for routine use should generally not occur and perhaps treated as professional misconduct.

**Table 3:** Free-text responses about who should provide independent approval if it is required to use a restricted antimicrobial.

Reference	Quote
3a	It needs to be a system with wide representation, veterinary and human health, not just one type.
3b	Similar to medical general practitioner – qualified and experienced public health veterinarian in CVO office to be contacted prior to antibiotic being prescribed.
3c	A team of experts including pharmacologist, microbiologist, internal medicine and/or surgery specialist, etc.
3d	Too practice and location specific. Must be practical. Ideally it's a veterinarian with expertise in infectious diseases. That might be university stewardship academics but not all those have the clinical expertise that is needed for proper case discussions.

**Table 4:** Free-text responses about the purpose of reporting the use of antimicrobials not registered for use in animals.

Reference	Quote
4a	High importance antibiotic use should be discouraged, though may play an important role in exceptional cases. Any use would need to be monitored and assessed for appropriateness.
4b	Documentation and surveillance needed to provide oversight, but should not be punitive to individual users.
4c	We need the surveillance to create data if our prescriptions drive resistance levels, and audit and education is better than regulation and punishing
4d	Would be great if this included the animal, indication etc. to identify trends and areas for targeted improvement
4e	I think the most important principle here is accountability for prescribers, and ensuring prescribers think through the rational for, and implications of, their decisions

## Data Availability

Data will be made available on request.

## Appendix A Supplementary data

Survey questions Image 1
