# Supplementary material for: Reaching consensus amongst international experts on the use of high importance-rated antimicrobials in animals – a Delphi study
Source: One Health. 2024 Aug 26;19:100883. doi: 10.1016/j.onehlt.2024.100883 (PMC11406009; doi:10.1016/j.onehlt.2024.100883)
Supplement: Supplementary file 1 — Survey questions [file mmc1.docx]

Supplementary Materials

**Delphi Survey Questions**

**Round 1**

Q1

"Do you prescribe antibiotics in your current role?"

Yes

No

Q2

"Are you the lead of an antimicrobial stewardship team, or do you have executive oversight over an antimicrobial stewardship team?"

Yes

No

Q3

"What is your occupation (select all that apply)?"

[Checked or Unchecked for each possible response]

Veterinarian

Physician/Surgeon

General practitioner

Pharmacist

Medical or Veterinary Microbiologist

Public Health physician/official

Government employee

Other

Please specify [free text box]

Q4 "What species do you predominately work with? (tick all that apply)"

Dog and cats

Horses

Beef cattle

Dairy cattle

Poultry

Exotics

Pigs

Sheep

Other

Q5 "Please select your area(s) of expertise (select all that apply)"

Infectious diseases

Microbiology

Antimicrobial stewardship

Antimicrobial resistance

Infection prevention and control

Medicine

Surgery

General practice

Public health

Other

Please specify [free text box]

Q6

"How many years have you been working in antimicrobial stewardship or antimicrobial resistance?"

< 5 years

5-10 years

11-19 years

>20 years

Q7 "What country do you primarily practice in?"

Afghanistan

Albania

Algeria

Andorra

Angola

Antigua & Deps

Argentina

Armenia

Australia

Austria

Azerbaijan

Bahamas

Bahrain

Bangladesh

Barbados

Belarus

Belgium

Belize

Benin

Bhutan

Bolivia

Bosnia Herzegovina

Botswana

Brazil

Brunei

Bulgaria

Burkina

Burundi

Cambodia

Cameroon

Canada

Cape Verde

Central African Rep

Chad

Chile

China

Colombia

Comoros

Congo

Congo {Democratic Rep}

Costa Rica

Croatia

Cuba

Cyprus

Czech Republic

Denmark

Djibouti

Dominica

Dominican Republic

East Timor

Ecuador

Egypt

El Salvador

Equatorial Guinea

Eritrea

Estonia

Ethiopia

Fiji

Finland

France

Gabon

Gambia

Georgia

Germany

Ghana

Greece

Grenada

Guatemala

Guinea

Guinea-Bissau

Guyana

Haiti

Honduras

Hungary

Iceland

India

Indonesia

Iran

Iraq

Ireland {Republic}

Israel

Italy

Ivory Coast

Jamaica

Japan

Jordan

Kazakhstan

Kenya

Kiribati

Korea North

Korea South

Kosovo

Kuwait

Kyrgyzstan

Laos

Latvia

Lebanon

Lesotho

Liberia

Libya

Liechtenstein

Lithuania

Luxembourg

Macedonia

Madagascar

Malawi

Malaysia

Maldives

Mali

Malta

Marshall Islands

Mauritania

Mauritius

Mexico

Micronesia

Moldova

Monaco

Mongolia

Montenegro

Morocco

Mozambique

Myanmar {Burma}

Namibia

Nauru

Nepal

Netherlands

New Zealand

Nicaragua

Niger

Nigeria

Norway

Oman

Pakistan

Palau

Panama

Papua New Guinea

Paraguay

Peru

Philippines

Poland

Portugal

Qatar

Romania

Russian Federation

Rwanda

St Kitts & Nevis

St Lucia

Saint Vincent & the Grenadines

Samoa

San Marino

Sao Tome & Principe

Saudi Arabia

Senegal

Serbia

Seychelles

Sierra Leone

Singapore

Slovakia

Slovenia

Solomon Islands

Somalia

South Africa

South Sudan

Spain

Sri Lanka

Sudan

Suriname

Swaziland

Sweden

Switzerland

Syria

Taiwan

Tajikistan

Tanzania

Thailand

Togo

Tonga

Trinidad & Tobago

Tunisia

Turkey

Turkmenistan

Tuvalu

Uganda

Ukraine

United Arab Emirates

United Kingdom

United States

Uruguay

Uzbekistan

Vanuatu

Vatican City

Venezuela

Vietnam

Yemen

Zambia

Zimbabwe

Q8

"In which state do you primarily work?"

ACT

NSW

NT

SA

QLD

TAS

VIC

WA

Q9 "Which antimicrobial importance rating system do you use in your clinical practice or workplace? (select all that apply)"

Practice/institution specific

Country-specific (e.g. ASTAG, BSAVA Protect Me traffic lights, Canadian categorization)

World Health Organistation

OIE (World Organisation for Animal Health)

Unsure

None

Other

Please specify [free text box]

Q10

"Veterinarians should be able to create practice specific rating systems to suit their situations. *For example, choose to reclassify cefovecin (a third generation cephalosporin) as lower importance."

Strongly agree

Somewhat agree

Neither agree nor disagree

Somewhat disagree

Strongly disagree

Q11 "In your view which rating systems should veterinarians use?"

Country-specific (i.e. ASTAG)

World Health Organistation

OIE (World Organisation for Animal Health)

Other 🡪Please specify [free text box]

Q12

"Is there anything else you'd like to tell us about rating systems for antimicrobial importance in veterinary medicine including anything about the implementation of such systems?"

[free text box]

Q13

"If restrictions were to be placed on veterinary prescribing of antimicrobials, which antimicrobials should they apply to? (select one)"

All antimicrobials

Antimicrobials with medium and high importance rating

All antimicrobials with high importance rating

All high-importance rating antimicrobials except 3rd generation cephalosporins and fluoroquinolones

All high-importance rating antimicrobials except fluoroquinolones

All high-importance rating antimicrobials except 3rd generation cephalosporins

No antimicrobials should be restricted

Q14 "If there were to be restrictions placed on veterinary prescribing of antimicrobials with high importance rating, which of the following do you think is appropriate?"

"These antimicrobials must not be used in veterinary medicine under any circumstance"

Strongly agree

Somewhat agree

Neither agree nor disagree

Somewhat disagree

Strongly disagree

"Use can only proceed with approval from an independent office"

Strongly agree

Somewhat agree

Neither agree nor disagree

Somewhat disagree

Strongly disagree

"Use in referral hospitals is allowed without approval"

Strongly agree

Somewhat agree

Neither agree nor disagree

Somewhat disagree

Strongly disagree

"Use in general practice requires approval from an independent office"

Strongly agree

Somewhat agree

Neither agree nor disagree

Somewhat disagree

Strongly disagree

"Use is allowed while waiting for culture and susceptibility results, if there is a high suspicion of need for use and the animal is critically ill"

Strongly agree

Somewhat agree

Neither agree nor disagree

Somewhat disagree

Strongly disagree

"Use is only allowed after culture and susceptibility testing confirms that the pathogen is resistant to all low and medium rated antimicrobials that could be used to treat the case"

Strongly agree

Somewhat agree

Neither agree nor disagree

Somewhat disagree

Strongly disagree

"Use is allowed after treatment failure with a lower importance antimicrobial"

Strongly agree

Somewhat agree

Neither agree nor disagree

Somewhat disagree

Strongly disagree

"Use is allowed in critically ill animals"

Strongly agree

Somewhat agree

Neither agree nor disagree

Somewhat disagree

Strongly disagree

Q15

"Should antimicrobials with high-importance to human medicine that are registered for use in veterinary medicine be treated differently to those that are not?

Examples of antimicrobials with high-importance that are registered for use in veterinary medicine include; 3rd generation cephalosporins, fluoroquinolones, virginiamycin and macrolides. Human formulations for which no registered veterinary product exist include; amikacin, imipenem and ticarcillin-clavulanate. "

Yes

Unsure

No

Q15

"How should these high-importance to human medicine antimicrobials that are registered for use in veterinary medicine be treated differently?"

[free text box]

Q16

"If independent approval is required to use a restricted antimicrobial, who do you think should provide this approval?"

Practice stewardship champion

Veterinary microbiologist

Corporate regional clinical director (where this role exists)

Office of the state chief veterinary officer

Australian Veterinary Association

University antimicrobial stewardship academics

Veterinary pharmacologist

Other

Q16

"If you selected 'other', please specify"

[free text box]

Q17

"Are there other restrictions you think should be placed on the prescribing of antimicrobials with high importance to human health?"

Yes

No

Q17

"What other restrictions do you think should be placed on antimicrobials with high-importance to human health?"

[free text box]

**Round 2**

Q1

"In light of the previous results, please state whether you agree with the following statement.

Veterinarians should be able to create local practice-specific antimicrobial use protocols, but should not be able to create their own practice-specific antimicrobial importance rating systems."

Agree

Disagree

"Please provide your reasoning for this choice."

[free text box]

Q2

"Do you agree that practice-specific antimicrobial use protocols are justified in the following

situations? - Specific disease presentations not covered by guidelines"

1. Strongly agree

2. Agree

3. Neither agree nor disagree

4. Disagree

5. Strongly disagree

"Do you agree that practice-specific antimicrobial use protocols are justified in the following

situations? - When local susceptibility data suggests resistance to first-line antimicrobial treatment"

1. Strongly agree

2. Agree

3. Neither agree nor disagree

4. Disagree

5. Strongly disagree

Q2Reasoning

"Please provide your reasoning for the above choices."

[free text box]

Q3

"Considering the response to the rating system question above and the information provided at the start of this survey, would you agree with the following statement?

The country-specific rating system should take precedence over any other rating system (e.g. WHO rating system) when veterinarians make decisions about antimicrobial prescribing choices."

1. Yes

0. No

"Please provide your reasoning for this choice."

[free text box]

Q4

"When using international prescribing guidelines e.g. International Society for Companion Animal Infectious Diseases (ISCAID), British Equine Veterinary Association (BEVA),

these should be adapted to accountfor the country-specific rating system."

1. Yes

0. No

"Please provide your reasoning for this choice."

[free text box]

Q5

"Considering the results above, if restrictions were to be placed on veterinary prescribing of antimicrobials, which antimicrobials should they apply to? (select one)"

1, All high-importance rating antimicrobials except 3rd generation cephalosporins and fluoroquinolones

2, Antimicrobials with medium and high importance rating

3, All antimicrobials with high importance rating

4, No antimicrobials should be restricted

"Please provide your reasoning for this choice."

[free text box]

Q6

"Considering the results provided, do you agree with the following statements? Use of high importance antimicrobials is allowed after culture and susceptibility testing confirms that the pathogen is resistant to all low and medium rated antimicrobials that could be used to treat the case"

1. Agree

2. Disagree

"Considering the results provided, do you agree with the following statements? - Use of high importance antimicrobials is allowed while waiting for culture and

susceptibility results, if there is a high suspicion of need for use and the animal is critically ill"

1. Agree

2. Disagree

"Considering the results provided, do you agree with the following statements? - The use of high importance antimicrobials in veterinary medicine should NOT be banned."

1. Agree

2. Disagree

"Please provide your reasoning for this choice."

[free text box]

Q7

"Considering the results provided, do you agree with the following statement? Any use of high importance antimicrobials that are not registered for use in animals e.g. vancomycin, amikacin or imipenem, must be reported to a central authority."

1. Yes

0. No

"Please provide your reasoning for this choice."

[free text box]

Q8

"Do you have any other comments or questions about the use of high importance antimicrobials in a veterinary setting?"

Q9

"Do you have any other comments or questions about this consensus-building process?"

**Round 3**

Q1

"What is your primary occupation? (select one)"

1. Academic

2. Epidemiologist

3. Government Employee

4. Employed in Industry/Pharmaceutical sector

5. Medical or Veterinary Microbiologist

6. Pharmacist

7. Physician/Surgeon

8. Physician/Surgeon and Microbiologist

9. Veterinarian

10. Veterinarian and Academic

11. Epidemiologist (including expertise in diseases in

humans and/or animals)

12. Other

Q2Country

"In which country do you primarily work?"

1, Afghanistan

2, Albania

3, Algeria

4, Andorra

5, Angola

6, Antigua & Deps

7, Argentina

8, Armenia

9, Australia

10, Austria

11, Azerbaijan

12, Bahamas

13, Bahrain

14, Bangladesh

15, Barbados

16, Belarus

17, Belgium

18, Belize

19, Benin

20, Bhutan

21, Bolivia

22, Bosnia Herzegovina

23, Botswana

24, Brazil

25, Brunei

26, Bulgaria

27, Burkina

28, Burundi

29, Cambodia

30, Cameroon

31, Canada

32, Cape Verde

33, Central African Rep

34, Chad

35, Chile

36, China

37, Colombia

38, Comoros

39, Congo

40, Democratic Republic of the Congo

41, Costa Rica

42, Croatia

43, Cuba

44, Cyprus

45, Czech Republic

46, Denmark

47, Djibouti

48, Dominica

49, Dominican Republic

50, East Timor

51, Ecuador

52, Egypt

53, El Salvador

54, Equatorial Guinea

55, Eritrea

56, Estonia

57, Ethiopia

58, Fiji

59, Finland

60, France

61, Gabon

62, Gambia

63, Georgia

64, Germany

65, Ghana

66, Greece

67, Grenada

68, Guatemala

69, Guinea

70, Guinea-Bissau

71, Guyana

72, Haiti

73, Honduras

74, Hungary

75, Iceland

76, India

77, Indonesia

78, Iran

79, Iraq

80, Ireland {Republic}

81, Israel

82, Italy

83, Ivory Coast

84, Jamaica

85, Japan

86, Jordan

87, Kazakhstan

88, Kenya

89, Kiribati

90, Korea North

91, Korea South

92, Kosovo

93, Kuwait

94, Kyrgyzstan

95, Laos

96, Latvia

97, Lebanon

98, Lesotho

99, Liberia

100, Libya

101, Liechtenstein

102, Lithuania

103, Luxembourg

104, Macedonia

105, Madagascar

106, Malawi

107, Malaysia

108, Maldives

109, Mali

110, Malta

111, Marshall Islands

112, Mauritania

113, Mauritius

114, Mexico

115, Micronesia

116, Moldova

117, Monaco

118, Mongolia

119, Montenegro

120, Morocco

121, Mozambique

Myanmar, {Burma}

122, Namibia

123, Nauru

124, Nepal

125, Netherlands

126, New Zealand

127, Nicaragua

128, Niger

129, Nigeria

130, Norway

131, Oman

132, Pakistan

133, Palau

134, Panama

135, Papua New Guinea

136, Paraguay

137, Peru

138, Philippines

139, Poland

140, Portugal

141, Qatar

142, Romania

143, Russian Federation

144, Rwanda

145, St Kitts & Nevis

146, St Lucia

147, Saint Vincent & the Grenadines

148, Samoa

149, San Marino

150, Sao Tome & Principe

151, Saudi Arabia

152, Senegal

153, Serbia

154, Seychelles

155, Sierra Leone

156, Singapore

157, Slovakia

158, Slovenia

159, Solomon Islands

160, Somalia

161, South Africa

162, South Sudan

163, Spain

164, Sri Lanka

165, Sudan

166, Suriname

167, Swaziland

168, Sweden

169, Switzerland

170, Syria

171, Taiwan

172, Tajikistan

173, Tanzania

174, Thailand

175, Togo

176, Tonga

177, Trinidad & Tobago

178, Tunisia

179, Turkey

180, Turkmenistan

181, Tuvalu

182, Uganda

183, Ukraine

184, United Arab Emirates

185, United Kingdom

186, United States

187, Uruguay

188, Uzbekistan

189, Vanuatu

190, Vatican City

191, Venezuela

192, Vietnam

193, Yemen

194, Zambia

195, Zimbabwe

"Over the past two survey rounds, consensus amongst participants has been reached on the following items relating to the use of high importance antimicrobials as rated by the ASTAG (Australian Strategic and Technical Advisory Group on AMR) system.

1. The country-specific rating system should take precedence over any other rating system (e.g. WHO rating system) when veterinarians make decisions about antimicrobial prescribing choices.

2. When using international prescribing guidelines e.g. International Society for Companion Animal Infectious Diseases (ISCAID), British Equine Veterinary Association (BEVA), these should be adapted to account for the country-specific rating system.

3. Veterinarians should be able to create local practice-specific antimicrobial use protocols, but should not be able to create their own practice-specific antimicrobial importance rating systems.

4. Use of high importance antimicrobials is allowed after culture and susceptibility testing confirms that the pathogen is resistant to all low and medium rated antimicrobials that could be used to treat the case.

5. The use of high importance antimicrobials in veterinary medicine should NOT be banned.

6. Any use of high importance antimicrobials that are not registered for use in animals e.g. vancomycin, amikacin or imipenem, must be reported to a central authority.

Q3

How important do you think it is to reduce high importance antimicrobial use in companion animals?"

1, Very Important

2, Important

3, Moderately Important

4, Slightly Important

5, Not Important

Q4

How important do you think it is to reduce high importance antimicrobial use in food-producing animals?

1, Very Important

2, Important

3, Moderately Important

4, Slightly Important

5, Not Important

Q5

"To which central authority in Australia should use in animals of high importance antimicrobials that are not registered for animal use (e.g. vancomycin, amikacin, or imipenem) be reported?

Please read the following information to assist you in answering the question.

The Australian Pesticides and Veterinary Medicines Authority (APVMA) is responsible for registration of all agricultural and veterinary chemical products into the Australian marketplace.

A newly formed One Health independent federal body would work to address issues such as AMR using a one health framework with the goal of improving the coordination of actions related to the health of people, animals and the environment, particularly in relation to disease prevention and control.

State and territory departments of health in Australia are generally responsible for ‘control of use’ legislation to monitor the use of schedule 8 medicines such as ketamine and opioids in veterinary practice as well as schedule 4 medicines and poisons, including antibiotics. Resourcing for these purposes varies across states.

State departments of agriculture have varying roles across the states but generally promote and support the agriculture sector to develop and add value to the economy in partnership with farmers, industry and communities. Focus on companion animal issues varies across the states but these sectors generally do not receive a high level of funding or support.

The Australian Chief Veterinary Officer (ACVO) is the primary representative of, and advisor to, the Australian Government on matters relating to Australia’s animal health status. The objective of the Office of the Australian Chief Veterinary Officer (OCVO) is to mitigate threats to the Australian economy, and the productivity of Australia’s animal-dependent industries. The OCVO provides policy coordination, strategic direction and leadership on animal issues of national significance.

The Australian Strategic and Technical Advisory Group on AMR (ASTAG) is a working group composed of fourteen representatives with expertise in human, animal, plant and environmental health. The current role of ASTAG is to provide expert advice on AMR-related issues, research priorities and implementation approaches to support Australia’s national antimicrobial resistance strategy."

1, Australian Pesticides and Veterinary Medicines Authority (APVMA)

2, A newly created independent federal body to address antimicrobial resistance using a One Health framework e.g. Centre for Disease Control type of organisation or a One Health central authority

3, State Department of Health

4, State Department of Agriculture

5, Office of the Chief Veterinary Officer (OCVO)

6, Australian Strategic and Technical Advisory Group (ASTAG) on AMR working group

7, Other

"If you selected 'other', to which authority do you think use should be reported?"

[free text box]

"Please provide your reasoning for the above choice."

[free text box]

Q6

"Given consensus on item number 6, "Any use of high importance antimicrobials that are not registered for use in animals e.g. vancomycin, amikacin or imipenem, must be reported to a central authority", the purpose of the reporting should be for the following reasons: (Select all that apply)"

Record keeping over time for surveillance of antimicrobial use

Auditing or investigating high or frequent users to assist users in finding ways to reduce unnecessary use (non-punitive)

Acting as a deterrent due to the additional reporting requirements

Other

Please specify [free text box]

"Please provide your reasoning for this choice"

[free text box]

Q7

"Given the consensus on item number 6, “Any use of high importance antimicrobials that are not registered for use in animals e.g. vancomycin, amikacin or imipenem, must be reported to a central authority”, which data should be reported? (Select all that apply)"

1, The species the antimicrobial has been prescribed for in this occurrence

2, Whether culture and sensitivity testing has been used previously to support the use

3, Any other justification for use e.g. supporting diagnostic tests, reasons other lower importance antimicrobials can not be used

4, Other - please specify

"Please specify"

[free text box]

"Please provide your reasoning for this choice"

[free text box]

Q8

"Please state whether you agree or disagree with the following statement:

If any high importance antimicrobials (as rated by the ASTAG rating system) are prescribed to animals, a clear indication for use and justification for antimicrobial choice, must be recorded in the medical history along with the dose rate given, route of administration, the duration and the time point for review of the condition and associated antimicrobial therapy."

1, Agree

2, Disagree

3, Other - please specify

"Please specify"

[free text box]

Q9

"Do you have any other comments or questions about the use of high importance antimicrobials in a veterinary setting?"

[free text box]

Q10Process

"Do you have any other comments or questions about this consensus-building process?"

[free text box]

If you would like your name to be published with these consensus items when results of the consensus process are reported, please click here [hyperlink to new survey] and record your name as you would like it to appear along with any relevant post-nominals e.g. MANZCVS, PhD.
